# Inactivation of Aerosolized Hepatitis A Viral Droplets on Food Contact Surfaces by Ultraviolet-Light-Emitting Diodes at 255 nm and 279 nm

**DOI:** 10.3390/foods14111899

**Published:** 2025-05-27

**Authors:** Breanna Polen, Ankit Patras, Brahmaiah Pendyala, Doris H. D’Souza

**Affiliations:** 1 Department of Food Science, University of Tennessee, Knoxville, TN 37996, USA; jnn688@vols.utk.edu; 2 Department of Food and Animal Sciences, Tennessee State University, Nashville, TN 37209, USA; apatras@tnstate.edu (A.P.); bpendyal@tnstate.edu (B.P.)

**Keywords:** bioaerosols, non-enveloped foodborne virus, ultraviolet light, inactivation

## Abstract

Hepatitis A viral outbreaks continue to occur. It can be transmitted through aerosolized droplets and thus can contaminate surfaces and the environment. Ultraviolet light emitting diode (UV-C LED) systems are used for inactivation of microbes, though research is needed to determine optimal doses for aerosolized HAV inactivation. This study evaluates the UV-C LED doses for the inactivation of aerosolized hepatitis A virus (HAV) deposited on stainless-steel and glass discs. HAV was aseptically deposited onto stainless-steel or glass discs (1.27 cm diameter) using a nebulizer within a chamber followed by treatments for up to 1.5 min with 255 nm (surface dose = 0–76.5 mJ/cm^2^) or 279 nm (surface dose = 0–8.1 mJ/cm^2^) UV-C LED. Plaque assays were used to enumerate infectious titers of recovered viruses and data from three replicates were statistically analyzed. The calculated linear D_10_-value (UV-C dose for a 1-log reduction in aerosolized deposits) for HAV by 255 nm UV-C LED was 47.39 ± 7.40 and 40.0 ± 2.94 mJ/cm^2^ (R^2^ = 0.94 and 0.91) and using 279 nm UV-C LED were 6.60 ± 0.27 and 5.57 ± 0.74 mJ/cm^2^ (R^2^ = 0.98 and 0.94) on stainless-steel and glass discs, respectively. The non-linear Weibull model showed δ (dose needed for a 1-log reduction in aerosolized HAV deposits) values for HAV of 29.69 ± 5.49 and 35.25 ± 15.01 mJ/cm^2^ by 255 nm UV-C LED (R^2^ = 0.99 and 0.92) and 6.67 ± 0.63 and 5.21 ± 1.25 mJ/cm^2^ by 279 nm UV-C LED (R^2^ = 0.98 and 0.95) on stainless-steel and glass discs, respectively. These data indicate that 279 nm UV-C LED showed higher efficiency for HAV inactivation than 255 nm UV-C LED, and that Weibull models were a better fit when tailing was observed. This study provides the inactivation data needed to aid in designing UV-C LED systems for delivering doses required to inactivate bio-aerosolized HAV deposits on stainless-steel and glass.

## 1. Introduction

Hepatitis A virus (HAV) is a human foodborne pathogen that causes severe gastrointestinal illness throughout the world. It belongs to the genus *Hepatovirus* in the *Picornaviridae* family that comprises genera such as *Poliovirus*, *Enteroviruses*, *Coxsackieviruses*, *Echoviruses*, and *Rhinoviruses* [1,2]. This virus has a 30 nm diameter capsid and a single-stranded, positive-sense 7.5 kb RNA, encoding only one open reading frame (ORF), bound on each end by a long 5′ and a short 3′ nontranslated RNA segment, that is non-enveloped when shed in feces [1,3]. While HAV strains belong to a single serological group, there are seven distinct genotypes (GI to GVII), with GI, GII, GIII, and GVII being recognized as primarily human isolates, while the GIV, GV, and GVI genotypes are simian isolates [4]. Genotypes GI, GII, and GIII are subsequently separated into subtypes A and B, with GIA outbreaks frequently reported worldwide, while genotype IB has been mainly reported in the Middle East and parts of South Africa [5,6].

Upon ingestion, HAV can survive in the acidic stomach environment and be delivered to the liver or excreted via feces [7]. The incubation period of HAV lasts between 15 and 50 days, averaging around 30 days, until there are waves of viremia and a large quantity of fecal viral shedding, with some salivary shedding without the development of symptoms [7]. Commercial HAV vaccines began to be available in 1995, which resulted in a decrease in the number of HAV outbreaks; however, naïve populations are susceptible to HAV infections [8].

HAV is reported to be environmentally stable and maintains stability at both high temperatures (up to 80 °C) and low pH environments (as low as a pH of 2) [1]. HAV is mainly transmitted/spread through the fecal–oral route. HAV illness spread can occur via consumption of contaminated food if the food is being prepared and/or handled by a person infected with HAV(and shedding virus), from cross-contamination via contact surfaces or upon close contact with infected individuals as well as bio-aerosolized droplets [8,9]. Proper hand hygiene is imperative to prevent transmission during food handling in food service locations. HAV poses a high public health concern with infectious doses as low as 10 particles [10]. Common foods linked to HAV outbreaks include shellfish, vegetables, ready-to-eat salads, ice cream, juices, breads, and raw or undercooked foods [11]. In the USA, as of 4 August 2023, 37 states reported a total of 423 deaths, 27,435 hospitalizations, and 44,903 cases [12]. In 2013, six cases of HAV were reported in New York City, USA among food handlers [9,13]. Research on novel approaches to prevent HAV transmission and outbreak spread is still needed.

In addition to commonly touched food-contact surfaces, bioaerosols, which denote particles in suspension within a liquid or gas, similar to small droplets in air, are also known to transmit pathogens, including the severe acute respiratory syndrome coronavirus 2 (SARS-CoV-2) that resulted in the COVID-19 pandemic [13]. Several factors, including size of particle in aerosols, airflow, surface properties, and humidity, play a role in viral transmission. Particles <5 μm can penetrate airways traveling below to the alveolar space, while particles <10 μm penetrate down the glottis in the human respiratory tract [14,15].

The importance of environmental disinfection was reiterated following the COVID-19 pandemic. Chemical disinfectants can aid in the inactivation of microorganisms, but continuous human exposure raises concerns of the risk of asthma and chronic obstructive pulmonary disease in humans, while disinfection-by-products (DBPs) can also cause environmental issues [16]. Furthermore, HAV (being a non-enveloped virus) is known to be quite resistant to several chemical disinfectants compared to SARS-CoV-2 (an enveloped virus), highlighting the need to investigate alternate inactivation methods.

Therefore, ultraviolet light (UV)-C-based inactivation treatments for environmental disinfection gained popularity. While the relatively recent literature reported that UV-C systems can inactivate foodborne viruses on surfaces [17,18], the required doses for the inactivation of aerosolized foodborne viruses need to be investigated/determined.

Aerosolized virus inactivation studies typically focus on respiratory viruses of human health concern [16]. In addition, research on UV-C-based treatments for the inactivation of aerosolized viruses used varied experimental conditions; thus, direct comparison of results becomes difficult [19]. Aerosolized bacteriophage MS2 (a single-stranded RNA virus) at 8 log PFU/mL was shown to be more susceptible to low pressure 254 nm UV-C light at a dose of 339–423 μJ/cm^2^ resulting in a 90% reduction than aerosolized double-stranded RNA phi6 and dsDNA phage T7 viruses that required 662–1190 μJ/cm^2^ for 90% reduction in infectious titers [20].

The application of UV-C light emitting diode (LED) systems of varying wavelength for microbial inactivation is being researched in order to overcome the disadvantages of traditional mercury lamp-based UV-C systems. Studies showed that 275 nm UV-C LED treatments could cause higher reduction of titers of SARS-CoV-2 (4.23 log TCID_50_ reduction after 5 s; corresponding dose of 399 µJ/cm^2^) than traditional 254 nm UV-C (1.17 log TCID_50_ reduction after 5 s; corresponding dose of 4250 µJ/cm^2^), or the excimer lamp at 222 nm (0.33 log TCID_50_ reduction after 5 s; corresponding dose of 35 µJ/cm^2^) [21]. Furthermore, treatments with UV-C LED at 279 nm at doses of 6 mJ/cm^2^ were reported to cause inactivation of aerosolized deposits of *E. coli*, *Pseudomonas fragi*, and *Salmonella* Enteritidis on stainless-steel, glass, and silicon rubber discs within a chambered setting by 2.80, 3.56, and 3.81 log CFU/mL, respectively [14].

UV-C LED systems using 279 nm wavelengths showed the ability to inactivate feline calicivirus and Tulane virus (both cultivable human norovirus surrogates), as well as HAV when surface spread and surface dried on ceramic, glass, Formica, and stainless-steel surfaces [17,18]. However, the use of 255 nm or 279 nm UV-C LED systems to inactivate aerosolized HAV deposits on these surfaces needs to be investigated. Additionally, the properties of bioaerosols that enable viral transmission need to be understood in order to determine and deliver optimal doses for inactivation. Therefore, the research objectives are to determine the doses of 255 nm and 279 nm UV-C LED systems required to inactivate bio-aerosolized droplets of HAV on glass, as well as stainless-steel discs (common contact surfaces associated with the food environment).

## 2. Materials and Methods

### 2.1. Animal Cell Culture as Hosts

The host cells, fetal rhesus monkey kidney cells (FRHK-4) obtained from the cell-culture collection at the University of Tennessee, Knoxville, were utilized for HAV infection and to allow for the propagation of hepatitis A virus (HAV-HM175) as previously reported in our studies [17,22,23]. Maintenance and propagation of viral host cell lines were carried out via the use of Dulbecco’s Modified Eagle Medium (DMEM-F12) that contained 1% Penicillin-Streptomycin (Pen-Strep) and 10% fetal bovine serum (FBS; Fisher Scientific, Pittsburgh, PA, USA).

### 2.2. Propagation of HAV

HAV strain-HM175 (obtained from/provided by Dr. Kniel’s laboratory at the University of Delaware) was propagated using previously described established protocols [17,18]. To briefly summarize, 2 mL of HAV was combined with confluent FRHK-4 cells contained in sterile 175 cm^2^ cell-culture flasks. Then, 8 mL DMEM-F12 along with 1.2% Pen-strep-2% FBS was added. These cells containing virus were incubated for 2.5 to 3 h at 37 °C in a CO_2_ incubator containing 5% CO_2_. After this incubation time, an additional 10 mL of DMEM-F12 with 1.2% Pen-strep and 10% FBS was added. The incubation at 37 °C proceeded for three to five days. Once cytopathic effects were noticeable, the infected flask was transferred to a −80 °C freezer and allowed to freeze. Then, the cells were thawed at room temperature and this process was repeated thrice. The supernatant from the third cycle of thawing was centrifuged for 10 min at 5000 rpm, passed through a 0.2 micron filter and this HAV filtrate (as stock, ~7 log PFU/mL) was then stored again at −80 °C as reported previously [17,18].

### 2.3. Aerosolization of HAV Droplets on Discs

Stainless-steel and glass discs (1.27 cm diameter) purchased from Biosurface Technologies Corporation (Bozeman, MT, USA) were surface washed with 70% ethanol and air-dried. The dried discs were wrapped in aluminum foil and autoclaved. These autoclaved discs were transferred to a sterile Petri dish. The Petri dish containing the discs were placed within a modified polyplastic chamber (27.94 × 27.94 × 27.94 cm^3^) with a centered circular cutout within the base of the chamber, placed on stainless-steel jack, and all kept within a biosafety hood as described in earlier studies [14,24]. An atomizer that was used in our previous studies (4 jet Blaustein Atomizing Module (BLAM) type, CH Technologies, Westwood, NJ, USA) was placed and balanced within the centered circular cutout and used for aerosolization/ nebulization of HAV [14,24]. A 0.5 mL of test suspension containing 0.25 mL of HAV stock (~7 log PFU) and 0.25 mL phosphate-buffered solution (PBS) was inserted at a rate of 700 µL/min into this nebulizer unit with a 1 mL syringe connected to an air compressor that supplied 25 psi of compressed air. Aerosolization of HAV droplets was conducted on discs in sets of 3 within a Petri dish; each set contained a 0 s exposure time disc for use as a control. The stainless-steel or glass discs were placed at 15.24 cm from the spraying ports directly below the nebulizer to generate bioaerosols of HAV, and the bioaerosols were allowed to settle on the surface before the nebulizer was replaced with the 255 nm or with the 279 nm UV-C LED systems through the cut-out in the designed chamber.

### 2.4. Aerosolized HAV Deposit Treatments with UV-C LED (255 nm or 279 nm) on Stainless-Steel and Glass Discs

As mentioned above, the 255 nm or 279 nm UV-C LED device was placed on top of the polyplastic chamber base centered at the circular cutout directly 15.24 cm above the HAV inoculated/deposited stainless-steel or glass disc. Treatments of HAV for up to 1.5 min with the 255 nm or 279 nm UV-C LED systems (MD1016-1, Irtronix, Torrence, CA, USA) [14,24] were carried out on glass and stainless-steel discs using 15 s intervals until 30 s followed by 60 s and resuming 15 s intervals until 90 s, including a control within each set of 3 discs (0 s, 15 s, 30 s, 0 s, 45 s, 60 s, 0 s, 75 s, 90 s) (10.414 cm in from sample, Voltage = 22–54, Ampere = 2.1 (max), surface dose = 0–76.5 mJ/cm^2^ for 255 nm UV-C LED; and was 10.414 cm from sample, Voltage = 27.1, Ampere = 0.1, surface dose = 0–8.1 mJ/cm^2^ for 279 nm UV-C LED).

### 2.5. Hepatitis A Virus Recovery

After treatment of the HAV inoculated discs was completed, these discs (as well as the control inoculated discs without UV-C treatment) were removed using sterile tweezers and aseptically placed within a sterile 15 mL centrifuge tube and then 1 mL DMEM-F12 with 1.2% Pen-Strep and 2% FBS was added and vortexed. The extracted HAV from each sample/surface was then ten-fold serially diluted using 2 mL centrifuge tubes containing 1.5 mL DMEM-F12 with 1.2% Pen-strep and 2% FBS. Recovered infectious viruses of treatment were then enumerated using plaque assays performed in duplicate, where treatment was repeated thrice.

### 2.6. HAV Infectious Plaque Assay

Confluent FRHK-4 host cells in sterile six-well cell-culture plates were used to enumerate infectious HAV by inoculation with 500 µL HAV recovered from the control or treated discs that was ten-fold serially diluted in the DMEM-F12 cell-culture media. The infected plates were kept for incubation in a CO_2_ incubator at 37 °C with 5% CO_2_ for two and a half hours. After this time, the media was aspirated and 2 mL containing a 1:1 ratio of 1.5% Noble agar and HAV overlay media was added per well [17,22]. The infected plates were stored in the same CO_2_ incubator at 37 °C with 5% CO_2_ for 72–120 h until visualization of plaques that were then enumerated and reported as log PFU/mL [17].

### 2.7. Dose Calculation of UV-C at 255 nm and 279 nm

The surface doses were calculated using the measured UV-C irradiance and various exposure/treatment times (secs) as carried out as described in our previous research [18]. The UV irradiance for the UV-C LED (279 nm) system was 0.09 mW/cm^2^ and the UV-C irradiance for the UV-C LED (255 nm) system was 0.85 mW/cm^2^ [17,18,25].

“Surface UV-C dosage (mJ/cm^2^) = UV intensity (mW/cm^2^) × exposure time (s)” (1)

### 2.8. Statistical Analysis of Recovered HAV

Treatments of bio-aerosolized HAV droplets using 255 nm and 279 nm UV-C systems at each time/dosage were replicated thrice on both stainless-steel and glass discs along with untreated viruses with zero seconds of UV-C exposure as control. Statistical analysis using a two-way ANOVA along with Tukey’s adjustments (*p ≤* 0.05; JMP v.17) was undertaken to determine significant differences of HAV inactivation between either surfaces, systems, and/or their interactions [18]. The D_10_-values from linear models were determined using Excel^®^, while Weibull models were used to determine non-linear fit using a GinaFit Tool (Excel Add-in). The model fit statistics and D_10_-values were compared among these two models.

A Weibull (non-linear) model was used to describe the viral infectivity when treated by UV-C light, with survival/infectivity curve shape determined with various parameters of distribution [26].

(2) $$log\left(N\right)=log\left(N_{0}\right)-(\frac{UV\;fluence}{\delta }{)}^{p}$$

“The incremental log reduction/doses were quantified using Equation (2), where N is the infectious virus count (PFU/mL) after treatment, N_0_ is the initial infectious virus count (PFU/mL), and UV fluence is expressed as mJ/cm^2^” [26].

Weibull models are reported to be desirable for microbial survival (viral infectivity) estimations during tailing effects after specific UV-C fluence treatments. The δ parameter signifies the first reduction time of the infectious virus, similar to that of the D_10_-value, as it refers to the dose needed to achieve 1-log microbial (infectious virus) reduction [26]. “*p* refers to the “shape parameter” that is connected to the shape of the produced curve (*p* < 1, convex curve, *p* > 1, concave curve, *p* = 1, straight line)” [27].

## 3. Results

### 3.1. Bioaerosol Deposited HAV Inactivation on Stainless-Steel Discs by a 255 nm UV-C LED System

**Figure 1:**
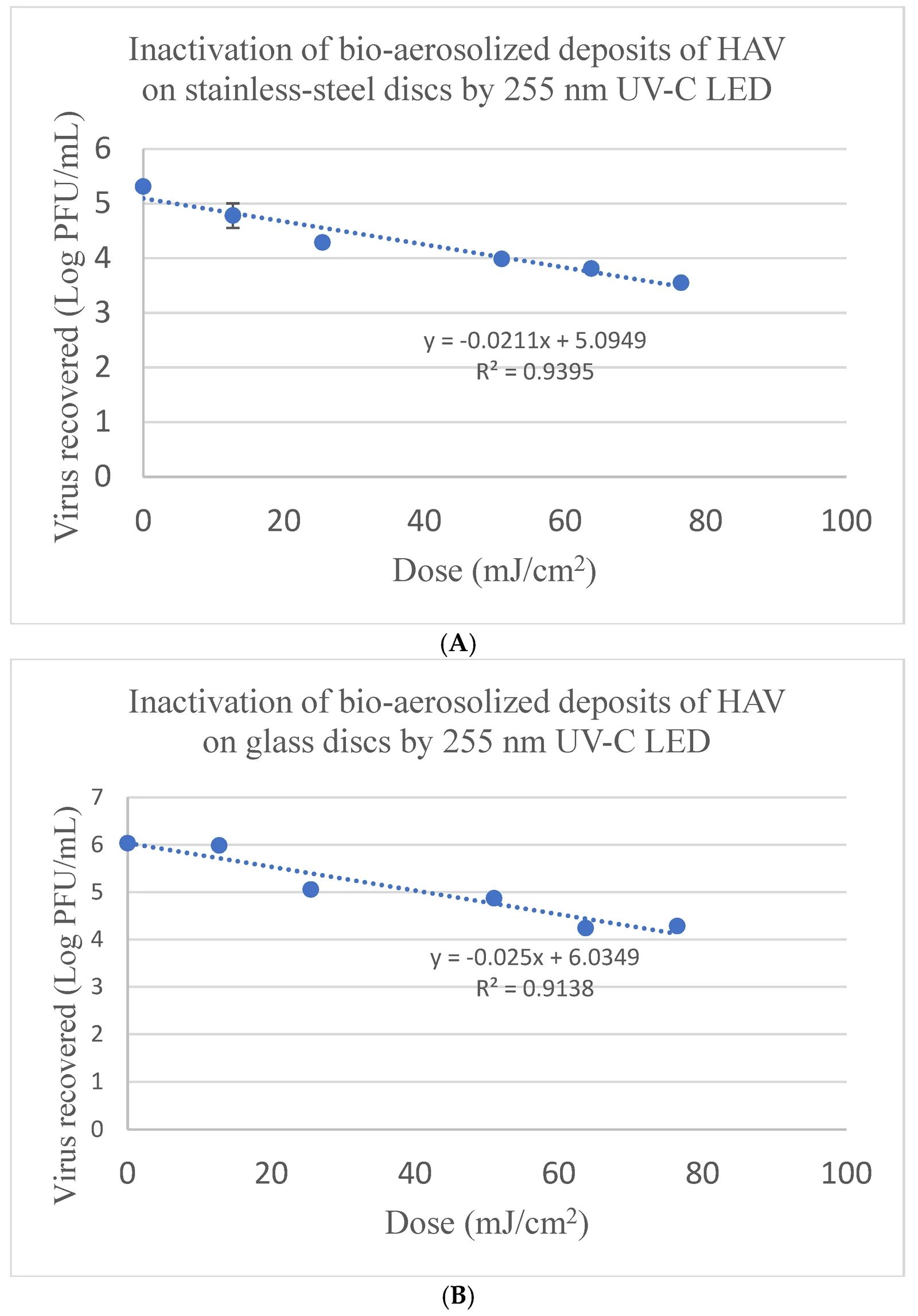
Linear model curves for the inactivation of bio-aerosolized deposits of HAV by UV-C LED at 255 nm on (**A**) stainless-steel surfaces and (**B**) glass surfaces. Corresponding Linear 1D value for HAV on stainless-steel surfaces = 47.39 mJ/cm^2^; Linear 2D = 94.79 mJ/cm^2^; Linear 3D = 142.18 mJ/cm^2^; and for HAV on glass discs linear 1D value = 40.0 mJ/cm^2^; Linear 2D = 80.0 mJ/cm^2^; Linear 3D = 120.0 mJ/cm^2^.

**Figure 2:**
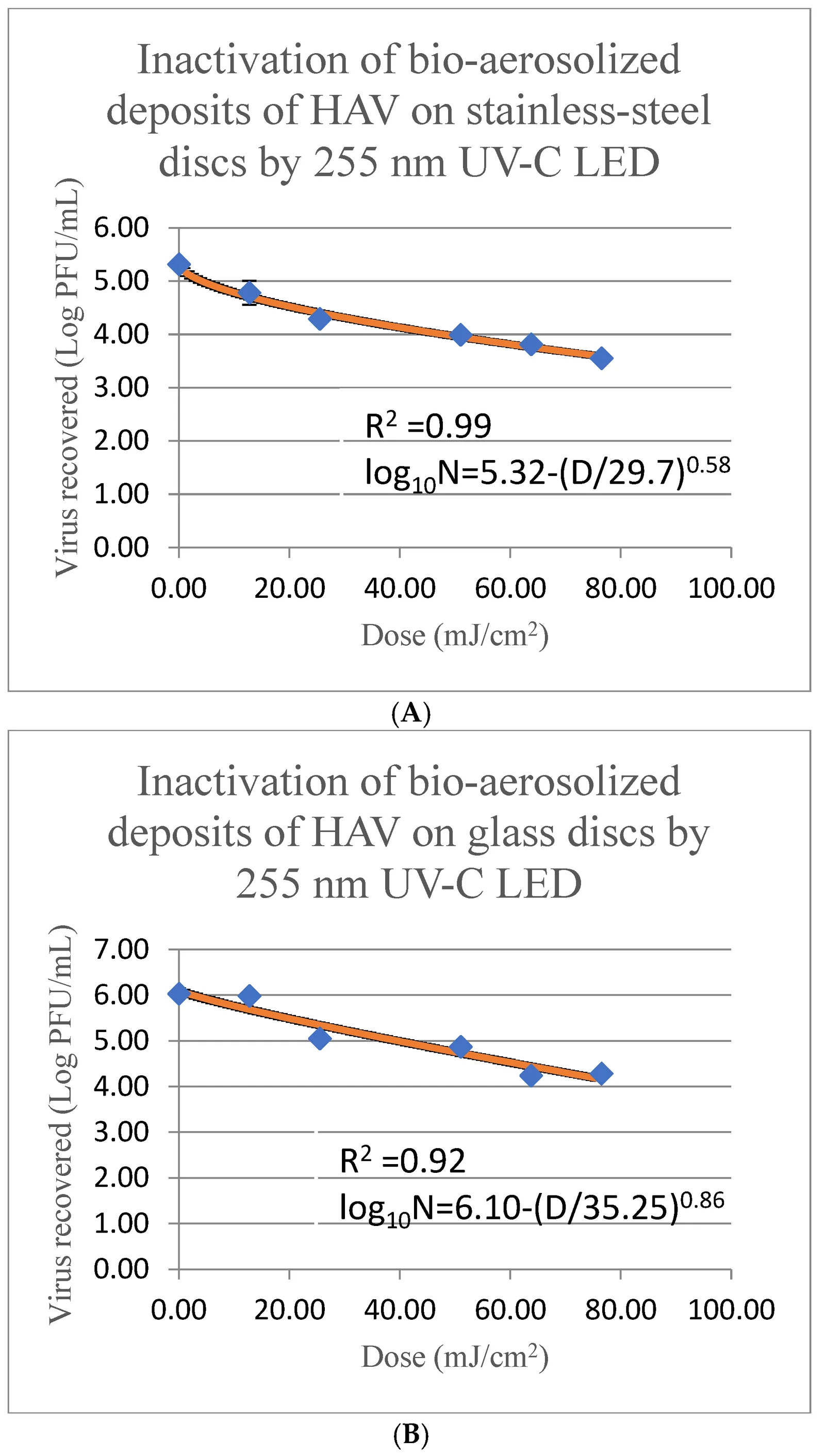
Weibull model fitted UV-C dose response curves of HAV inactivation on (**A**) stainless-steel disc surfaces and (**B**) glass disc surfaces using UV-C LED at 255 nm. Corresponding Weibull model 1D value for HAV on stainless-steel surfaces = 29.69 mJ/cm^2^; Weibull 2D = 97.82 mJ/cm^2^; Weibull 3D = 196.47 mJ/cm^2^; and on glass discs Weibull 1D value = 35.25 mJ/cm^2^; Weibull 2D = 79.13 mJ/cm^2^; Weibull 3D = 126.99 mJ/cm^2^.“Where ‘N’ is the initial concentration of the target virus, ‘N0’ is the infectious units of the virus after the dose, the Weibull slope (or threshold parameter) is depicted by ‘p’, ‘δ’ represents 90% reduction in target viral populations, ‘D’ depicts the exposure dosage of the target microorganism. The numerical values for inactivation kinetics parameters, as well as the dose needed to obtain 5-log reduction, and other related parameters were calculated for the model. The values of δ and *p* were used to determine a targeted log reduction using a specific dose. The dose required to obtain an x log reduction (txd) of the target microorganism was calculated as reported in literature” [24].

The linear model D_10_-value (mJ/cm^2^), or the dose required to result in a 1-log reduction in aerosolized deposits of HAV on stainless-steel disc surfaces by 255 nm UV-C LED was 47.39 ± 7.40 mJ/cm^2^ as shown in Table 1 and Figure 1A. The shape and scale parameters of the Weibull model inactivation curves were quantified and represented in Table 1 (Figure 2A). The Weibull model showed that a dose of 29.69 ± 5.49 mJ/cm^2^ (δ) was needed for a 1-log reduction in aerosolized deposits of HAV on stainless-steel discs using 255 nm UV-C LED as shown in Figure 2A. The Weibull model had a R^2^ value of 0.99, which was higher than that of the linear model (R^2^ value of 0.94), with a shape parameter (*p*) of 0.58 (Table 1).

Averages of triplicate treatments ± standard deviations are represented; please also note that the UV intensities are different between the two optical devices.

Treatments of aerosolized deposits of HAV on stainless-steel discs with UV-C LED at 255 nm resulted in infectious titer reduction between 0.53 log PFU/mL after 0.25 min and 1.76 log PFU/mL after 1.5 min exposure to the system (corresponding to doses between 12.75–76.5 mJ/cm^2^), respectively (Table 2). Our data indicate that significant reductions in the recovered HAV titers were obtained (*p* ≤ 0.05) with decreases of 1.02 ± 0.04 log PFU/mL from the recovered control HAV titers (recovered titers from untreated surfaces) after treatment of 0.5 min (dose of 25.5 mJ/cm^2^) and was further decreased by 1.5 ± 0.02 log PFU/mL after exposure of 1.25 min (dose of 63.75 mJ/cm^2^) to the system.

### 3.2. Bioaerosol Deposited HAV Inactivation on Glass Discs by a 255 nm UV-C LED System

The linear model D_10_-value (mJ/cm^2^), or the dose required to achieve a 1-log reduction in aerosolized deposits of HAV on glass disc surfaces using UV-C LED at 255 nm was calculated to be 40.0 ± 2.94 mJ/cm^2^ as shown in Table 1 and Figure 1B. The Weibull model showed a 1-log reduction in aerosolized droplets of HAV on glass disc surfaces at a dose of 35.25 ± 15.01 mJ/cm^2^ (δ) (Table 1 and Figure 2B). The Weibull model displayed a shape parameter (*p*) of 0.86 and an R^2^ value of 0.92 while the linear model showed an R^2^ of 0.91 (Table 1).

Treatments of aerosolized deposits of HAV on glass disc surfaces with the 255 nm UV-C LED system resulted in decreases in infectious titers between 0.04 log PFU/mL (negligible) and 1.79 log PFU/mL after exposure of 0.25 min to 1.5 min to the system (dose range of 12.75–76.5 mJ/cm^2^), respectively (Table 3). Significant reductions in HAV titers were also obtained (*p* ≤ 0.05) with decreases by 0.97 ± 0.03 log PFU/mL when compared to the recovered virus titers from the non-treated control discs after 0.5 min treatment (corresponding to a dose of 25.5 mJ/cm^2^) and were further decreased by 1.79 ± 0.07 log PFU/mL after a 1.25 min exposure (corresponding to a dose of 63.75 mJ/cm^2^).

### 3.3. Inactivation of Bioaerosol Deposited HAV on Stainless-Steel Discs by a 279 nm UV-C LED System

**Figure 3:**
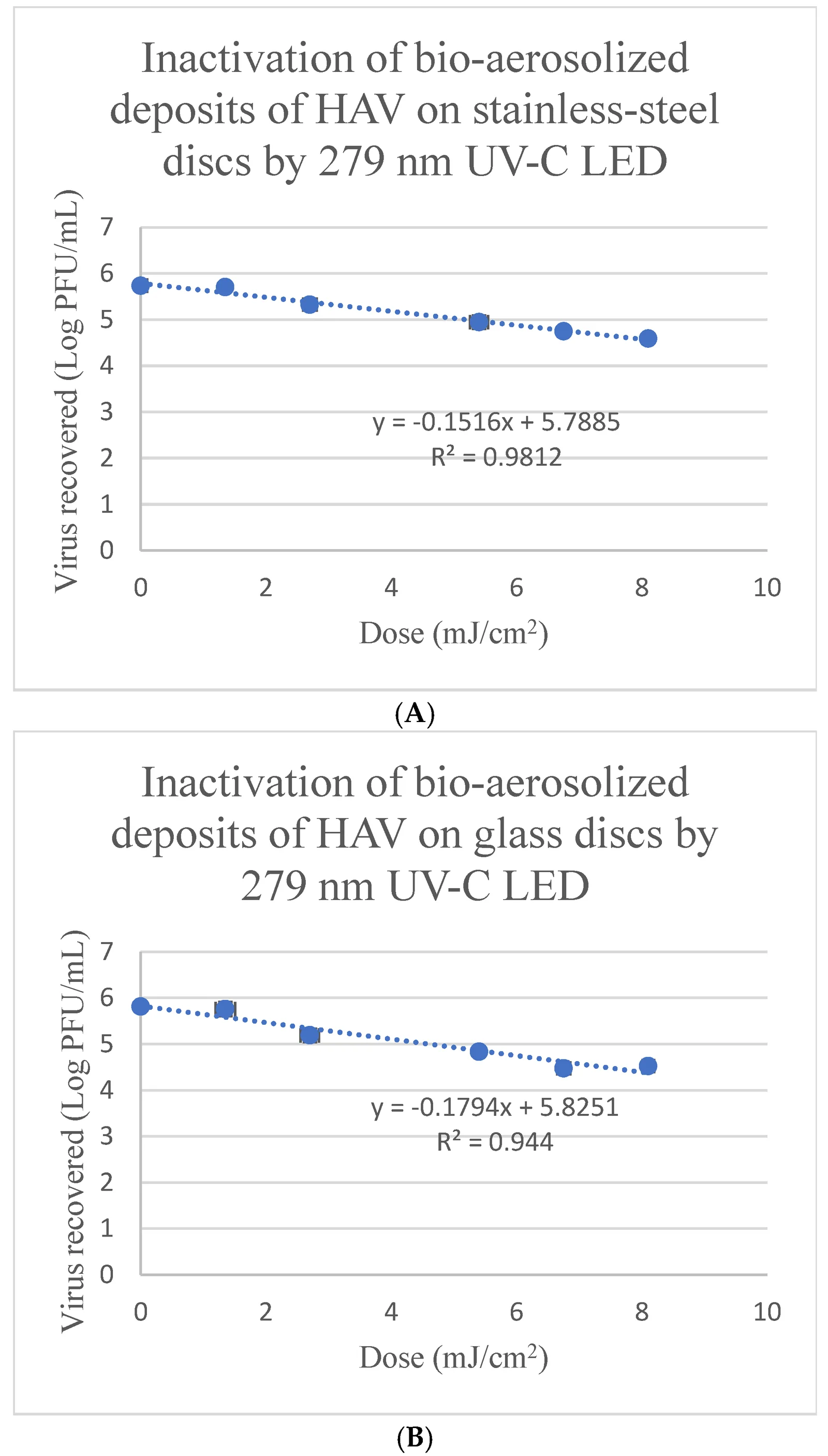
Linear model curves for the inactivation of bio-aerosolized deposits of HAV by UV-C LED at 279 nm on (**A**) stainless-steel disc surfaces and (**B**) glass disc surfaces. Corresponding Linear 1D value for HAV on stainless-steel disc surfaces = 6.60 mJ/cm^2^; Linear 2D = 13.19 mJ/cm^2^; Linear 3D = 19.79 mJ/cm^2^; and on glass disc surfaces linear 1D value = 5.57 mJ/cm^2^; Linear 2D _=_ 11.15 mJ/cm^2^; Linear 3D = 16.72 mJ/cm^2^.

**Figure 4:**
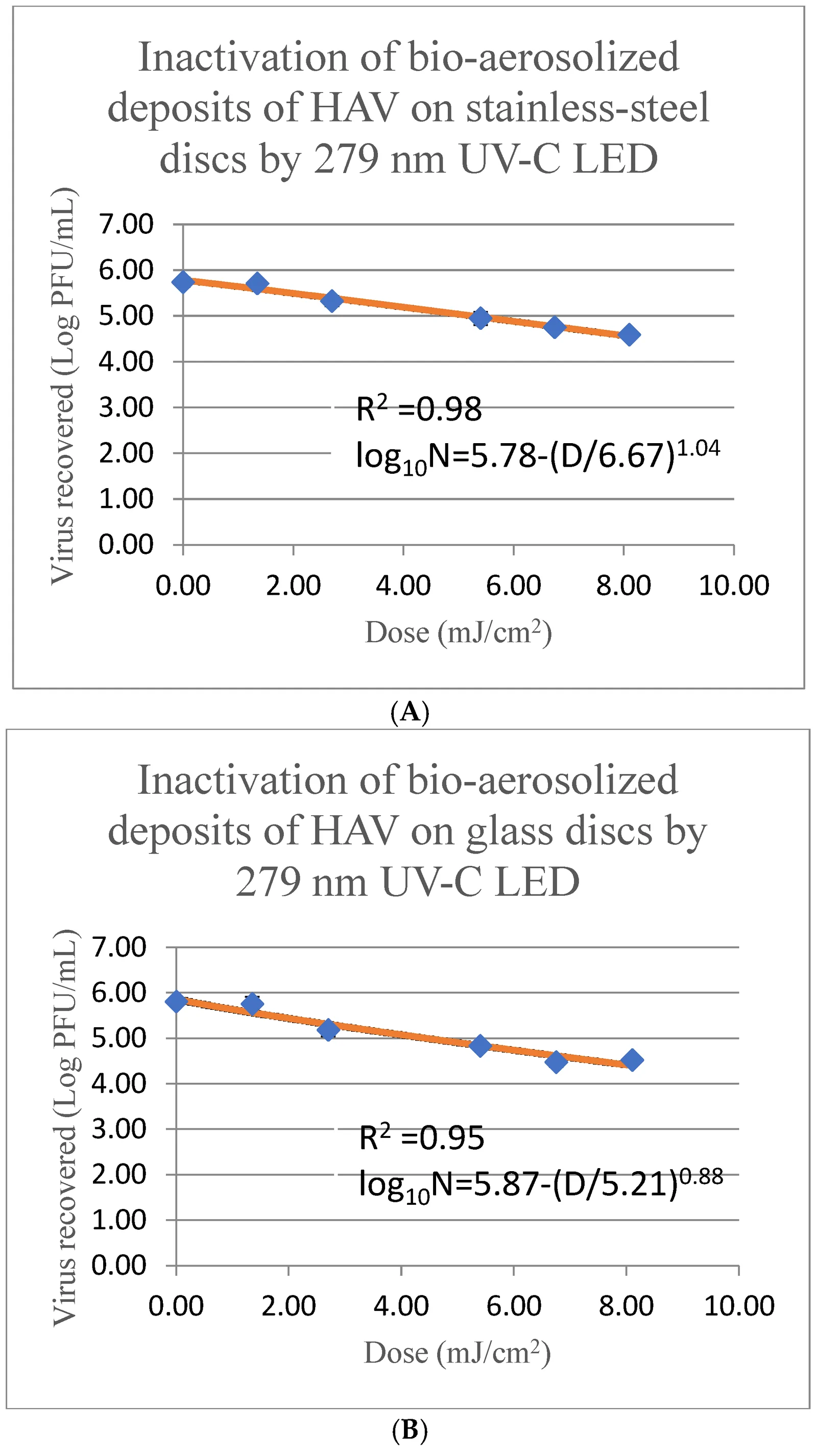
Weibull model fitted UV-C dose response curves of HAV inactivation on (**A**) stainless-steel disc surfaces and (**B**) glass disc surfaces using UV-C LED at 279 nm. Corresponding Weibull model 1D value for HAV on stainless-steel surfaces = 6.67 mJ/cm^2^; Weibull 2D = 12.98 mJ/cm^2^; Weibull 3D = 19.16 mJ/cm^2^; and on glass discs Weibull 1D value = 5.21 mJ/cm^2^; Weibull 2D = 11.49 mJ/cm^2^; Weibull 3D = 18.25 mJ/cm^2^.

The linear D_10_-value (mJ/cm^2^) for the inactivation of aerosolized deposits of HAV was 6.60 ± 0.27 mJ/cm^2^ (as shown in Table 1 and Figure 3A), while the Weibull model shows that a dose of 6.67 ± 0.63 mJ/cm^2^ (δ) was required for a 1-log reduction in aerosolized droplets of HAV on stainless-steel discs using the 279 nm UV-C LED system (Table 1 and Figure 4A). Both the linear and Weibull models R^2^ values were calculated to be 0.98, with the Weibull model displaying a shape parameter (*p*) of 1.04 (Table 1).

Treatments of aerosolized deposits of HAV on stainless-steel disc surfaces with UV-C LED at 279 nm resulted in decreases in infectious titers between 0.01 log PFU/mL (negligible) to 1.14 log PFU/mL after 0.25 min to 1.5 min exposure to the system (corresponding dose range of 1.35–8.1 mJ/cm^2^), respectively (Table 2). Significant reductions in HAV titers (*p* ≤ 0.05) by 0.41 log PFU/mL in comparison to the recovered titers from the non-treated inoculated control disc after treatment of 0.5 min (dose of 2.7 mJ/cm^2^) were observed. These HAV titers were further decreased by 0.78 log PFU/mL after an increase in treatment time to 1 min (corresponding to a dose of 5.4 mJ/cm^2^) and were still further decreased by 1.14 log PFU/mL after the longest exposure/treatment time used in this experiment of 1.5 min (corresponding to a dose of 8.1 mJ/cm^2^) for this virus in the current study.

### 3.4. Inactivation of Bioaerosol Deposited HAV on Glass Discs with a 279 nm UV-C LED System

The linear D_10_-value (mJ/cm^2^) to achieve a 1-log reduction in aerosolized deposits of HAV on glass disc surfaces was 5.57 ± 0.74 mJ/cm^2^ (as shown in Table 1, Figure 4A), while the Weibull model showed that a dose of 5.21 ± 1.25 mJ/cm^2^ (δ) was required for a 1-log decrease in titers of aerosolized droplets of HAV on glass disc surfaces using the 279 nm UV-C LED system (Table 1 and Figure 4B). The Weibull model with a shape parameter (*p*) of 0.88 showed a R^2^ value of 0.95 in comparison to the linear model R^2^ value of 0.94 (Table 1).

Treatments of aerosolized deposits of HAV on glass disc surfaces with the 279 nm UV-C LED system resulted in decreases in infectious titer of HAV between 0.05 log PFU/mL (negligible) to 1.29 log PFU/mL after exposure of 0.25 min to 1.5 min (dose range of 1.35–8.1 mJ/cm^2^), respectively (Table 3). Significant decreases in HAV titers (*p* ≤ 0.05) by 0.62 ± 0.15 log PFU/mL were observed in comparison to the titers from the untreated control after 0.5 min treatment (dose of 2.7 mJ/cm^2^). These titers were further decreased by 1.34 ± 0.10 log PFU/mL after 1.25 min treatment (dose of 6.75 mJ/cm^2^).

## 4. Discussion

As reported in the literature, pathogenic microorganisms can be spread through bioaerosols via infected individuals during talking, coughing, or sneezing, as well as during episodes of vomiting that require rapid and effective disinfection and decontamination strategies to decrease outbreak risk. For inactivation of pathogens in bioaerosols, ultraviolet (UV-C) technology has been investigated. However, the precise doses of novel UV-C LED devices for pathogen inactivation need to be determined [28].

Some recent studies with bioaerosols of bacteria, including *E. coli*, *P. fragi*, and *S.* Enteritidis (at 5.5 log CFU/mL), deposited on glass surfaces (1.27 cm diameter) reported that exposure to a dose of 6 mJ/cm^2^ of 279 nm UV-C LED could cause inactivation of 2.80, 3.56, and 3.81 log CFU/mL, respectively [14]. Other researchers studied bacterial aerosols of *L. monocytogenes*, *S. aureus*, *S.* Typhimurium, and *E. coli* and showed that 279 nm UV-C doses between 1.5 and 4.6 mJ/cm^2^ were needed for 2.5 log CFU/mL to 4.5 log CFU/mL reduction [29]. It was also shown that higher 279 nm UV-C doses of 23 mJ/cm^2^ were needed for the inactivation of 4-log fungal bioaerosols of *Aspergillus flavus* and *Al. japonica* compared to the lower doses needed for the tested bacteria [29]. Thus, inactivation rates are higher for bacteria than fungi, where bacteria show higher UV-C susceptibility, dependent on their size, cell structure, and other characteristics [29].

In relation to viruses, aerosolized bacteriophages such as MS2, QB, QX174 that are commonly used as enteric virus surrogates were shown to be inactivated by >4-log PFU/mL with 279 nm UV-C treatments at a dose of 4.6 mJ/cm^2^ [29]. Other studies with aerosolized viruses, including influenza A virus (H1N1 PR-8) as well as human coronaviruses (alpha HCoV-229E and beta HCoV-OC43), reported their D_10_-values to be 1.28 mJ/cm^2^ after exposure to far-UV-C at 222 nm, while treatment of influenza A virus (H1N1, PR-8) with the traditional 254 nm UV-C light system showed a slightly lower D_10_-value of 1.04 mJ/cm^2^ [30].

SARS-CoV-2 was shown to be inactivated by 3 log PFU/mL (assayed using Vero cells) by traditional 254 nm UV-C light systems at a dose of 3.7 mJ/cm^2^, while deep UV-LED at 280 nm exposure to a dose of 37.5 mJ/cm^2^ showed >3-log PFU/mL inactivation [31,32]. Similarly, other researchers showed that SARS-CoV-2 deposited on stainless-steel, plastic, and glass surfaces were significantly reduced in infectious titers by 3 log PFU/mL when treated with a pulsed broad-spectrum UV (200–700 nm) system at a dose of 17.2 mJ/cm^2^ [33]. UV-C light at 254 nm was shown to completely block aerosol transmission of SARS-CoV-2 among hamsters after treatments of the environment with a 21.4 mJ/cm^2^ dose of UV-C [34].

Taken together, the existing literature on the application of UV-C systems shows promise for the inactivation of bio-aerosolized bacteria, fungi, and viruses on surfaces. However, studies to determine the doses for the inactivation of aerosolized HAV are lacking, and these studies are needed because human enteric viruses can be transmitted via aerosolized vomitus. In the current study, UV-C LED systems at 255 nm and 279 nm for the inactivation/reduction in infectious titers of bio-aerosolized HAV deposited onto stainless-steel and glass were investigated when kept within a closed chamber. Bioaerosols of HAV deposited on stainless-steel discs and glass discs and treated with 279 nm UV-C LED showed maximum inactivation/infectious titer reduction of 1.14 log PFU/mL and 1.29 log PFU/mL at UV-C doses of 8.1 mJ/cm^2^ (Table 2), while HAV bioaerosols deposited on glass disc and stainless-steel surfaces after exposure to 255 nm UV-C LED resulted in maximum inactivation/infectious titer reduction of 1.75 ± 0.07 log PFU/mL and 1.76 ± 0.05 log PFU/mL at UV-C doses of 76.5 mJ/cm^2^ (Table 2 and Table 3). These results indicate higher dose requirements for the inactivation of HAV compared to the data previously reported and published for bacteria, under the same conditions [14]. However, previous researchers showed lower inactivation levels were obtained on silicon rubber surfaces, as well as stainless-steel (1.27 cm diameter) surfaces (although still significant at *p* ≤ 0.05), compared to glass surfaces, with 2.03 log PFU/mL, 3.14 log PFU/mL, and 3.03 log CFU/mL reduction for *E. coli*, *P. fragi*, and *Salmonella* on stainless steel, and 1.91 log PFU/mL, 3.08 log PFU/mL, and 2.91 log CFU/mL reduction for silicon rubber surfaces using the same doses (6 mJ/cm^2^ and UV-C LED at 279 nm) and systems [14]. This implies that surface types may play a role in the inactivation of bio-aerosolized bacterial deposits. This must be considered when designing optimal inactivation approaches. However, in the current study, similar levels of inactivation for treatments of HAV on stainless steel and glass were observed, with no significant differences between surface type for each tested system, as depicted in Table 1. Since stainless-steel and glass surfaces reportedly have similar porosity and surface roughness characteristics, the lack of significant differences in HAV inactivation when deposited on these surfaces could be attributed to these factors [18].

The current study shows that the 255 nm UV-C LED doses required to result in a 1-log decrease (D_10_-values) of aerosolized deposits of HAV on stainless-steel and glass disc surfaces were 47.39 ± 7.4 and 40.0 ± 2.94 mJ/cm^2^, respectively. Lower UV-C doses at 254 nm and UV-C LED at 279 nm were previously reported for the inactivation of HAV when HAV is surface-spread and dried on glass, Formica, stainless-steel, and ceramic surfaces. The D_10_-values of 9.49 ± 0.34 mJ/cm^2^, 7.26 ± 1.93 mJ/cm^2^, 9.49 ± 2.52 mJ/cm^2^, and 12.40 ± 1.15 mJ/cm^2^ were obtained on stainless steel, glass, ceramic, and Formica, respectively, when treated with 254 nm UV-C (mercury lamps) [17,18]. However, the linear model does not provide the parameters of optimal fit in the case of aerosolized deposits of HAV (R^2^ values of 0.94 and 0.91 for stainless steel and glass, respectively). The Weibull model provides a better fit (R^2^ values of 0.99 and 0.92 for stainless steel and glass, respectively) with (δ) values of 29.69 ± 5.49 and 35.25 ± 15.01 mJ/cm^2^, respectively. These differences in doses could be attributed to the tailing effects and non-linearity of the curves, indicating that the linear model may not be the best fit for determining the doses when tailing effects are observed.

The reported D_10_-values were 19.72 ± 2.45 mJ/cm^2^, 9.12 ± 2.08 mJ/cm^2^, 26.05 ± 0.60 mJ/cm^2^, and 12.39 ± 0.70 mJ/cm^2^ for HAV surface spread and dried on stainless steel, glass, ceramic, and Formica using UV-C LED at 279 nm, respectively [17,18]. The dose requirements (D_10_-value) from the current study for treatments of bio-aerosolized HAV deposited on glass surfaces and stainless-steel surfaces by the 279 nm UV-C LED system were 5.57 ± 0.74 mJ/cm^2^ and 6.60 ± 0.27 mJ/cm^2^, while the Weibull model showed that doses of 5.21 ± 1.25 mJ/cm^2^ and 6.67 ± 0.63 mJ/cm^2^ were needed to result in a 1-log reduction on glass disc surfaces and stainless-steel disc surfaces, respectively. The Weibull model with R^2^ values of 0.98 and 0.95 provided a better/improved fit compared to the linear model that had R^2^ values of 0.98 and 0.94 for HAV inactivation by the 279 nm UV-C system on stainless-steel and glass, respectively, as was also the case for the 255 nm UV-C LED system. The inactivation curves for HAV on glass disc surfaces and stainless-steel disc surfaces resulted in convex curves (*p* < 1.0) when fitted by the Weibull model, indicating there was an increasing rate of inactivation. The δ values indicate the estimated dose that is needed for the first log reduction, and this value increased as there was an increase in the *p* (shape-parameter) value [26].

These data suggest that higher doses are needed to inactivate surface dried HAV on glass surfaces and stainless-steel surfaces by the 279 nm UV-C LED system compared to aerosolized deposited HAV on these same surfaces [18]. For surface-dried HAV, 2.75 and 2.2 log PFU/mL reductions at UV-C doses of 49.2 and 19.68 mJ/cm^2^ were achieved, while bio-aerosolized droplet treatments showed maximum HAV titer reductions of 1.14 and 1.29 log PFU/mL at a UV-C dose of 8.1 mJ/cm^2^, though tailing effects were observed [18]. Hence, the data should be interpreted with caution taking into consideration both the linear and Weibull model data for determination of doses.

Furthermore, when comparing inactivation of viruses, it must be noted that enveloped viruses are more sensitive to UV-C treatments than non-enveloped viruses such as HAV. The increased susceptibility of enveloped viruses (such as SARS-CoV-2 and viruses that cause respiratory illnesses) are associated with their ability to undergo conformational changes with their lipid membranes on the outer surface of the capsid, which can result in loss of fusion proteins, while non-enveloped viruses can retain these fusion proteins on their strong and rigid capsid [35]. The previous literature also shows that HAV required increased doses for inactivation (compared to enveloped viruses) on dried surfaces due to its notably increased environmental and genetic resistances, including its VP4 protein within infectious particles, which is known to be targeted for viral neutralization [18,36,37]. Therefore, the higher dose requirements for a 1-log decrease in infectious titers of deposited bioaerosols of HAV on glass surfaces and stainless-steel surfaces with the 255 nm UV-C LED system is not surprising.

The differences between dry and wet viruses deposited onto surfaces could play a role in the increased dose requirements for the treatment of bio-aerosolized wet viral deposits, though evidence is currently lacking in this regard. *E. coli* on wet surfaces was shown to have a stronger resistance to treatments with the traditional UV-C at 254 nm in comparison to treatments on dry surfaces, indicating the liquid’s ability to absorb and block some UV-C irradiance [38]. Other factors to consider during the inactivation of bio-aerosolized pathogens include that during bio-aerosolization, various droplet sizes can be dispersed within the chamber, as well as within the Petri dishes containing these tested surfaces, allowing for potential overlap/accumulation of viral droplets underneath the surfaces, preventing penetration of UV-C.

In summary, this research determines the calculated linear model and Weibull model doses for 1-log inactivation of bio-aerosolized deposited forms of HAV on glass surfaces and stainless-steel surfaces (used as model food contact surfaces) by 255 nm UV-C LED and 279 nm UV-C LED systems. This study provides information that can be applied to design optimal UV-C LED systems for HAV inactivation on food contact surfaces when in bio-aerosolized form, depending on the model. However, even though this study provides the baseline dosage requirements for the inactivation of aerosolized HAV on stainless-steel and glass disc surfaces, it is important to note that higher doses may be needed in real-world scenarios that include contamination by aerosolized hepatitis A virus within organic matter, fecal matter or vomitus that warrants further investigation.

## 5. Conclusions

This study demonstrates the efficacy of 255 nm and 279 nm UV-C LED systems for inactivating bioaerosol-deposited hepatitis A virus (HAV) on glass and stainless-steel surfaces. The systems can be scaled up for use in the food and service industry. Higher UV-C doses at 279 nm were required to inactivate HAV in bioaerosol droplets compared to surface-dried HAV, with even greater doses needed at 255 nm. This research also shows that the inactivation of bio-aerosolized deposits of HAV was independent on the two tested surface types where the doses for inactivation on glass surfaces and stainless-steel surfaces were similar. However, the limitation of this study is that tailing effects were observed with the UV-C doses used and hence the data should be interpreted with caution when using log-linear models. The Weibull model was fitted to the experimental data and exhibited R^2^ > 0.85. These findings support the use of UV-C LED systems for viral inactivation in high-risk settings prone to cross-contamination from bio-aerosolized vomitus. Further research is warranted to optimize dosing strategies for HAV and other viruses within organic matrices, such as fecal matter or vomitus, to enhance outbreak prevention.

## Figures and Tables

**Table 1:** Comparison of the linear model and Weibull model parameters for the inactivation of bio-aerosolized HAV deposited on stainless-steel (SS) and glass discs by 255 nm and 279 nm UV-C LED systems.

	UV-C LED 255 nm					UV-C LED 279 nm				
	Linear		Weibull			Linear		Weibull		
HAV	D_10_-Value	R^2^	δ	*p*	R^2^	D_10_-Value	R^2^	δ	*p*	R^2^
SS	47.39 ± 7.40 ^Ba^	0.94	29.69 ± 5.49 ^Ba^	0.58	0.99	6.60 ± 0.27 ^Aa^	0.98	6.67 ± 0.63 ^Aa^	1.04	0.98
glass	40.0 ± 2.94 ^Ba^	0.91	35.25 ± 15.01 ^Ba^	0.86	0.92	5.57 ± 0.74 ^Aa^	0.94	5.21 ± 1.25 ^Aa^	0.88	0.95

δ: The dose needed for a 1-log viral reduction. Capital letters depict statistically significant differences during comparison between doses required for HAV inactivation by the two UV-C systems across one row within each model (*p ≤* 0.05). Lowercase letters depict statistically significant differences during comparison of doses for HAV inactivation between the two surfaces within one column (*p* ≤ 0.05).

**Table 2:** Inactivation of bio-aerosolized deposited HAV treated with either UV-C LED (255 nm) or UV-C LED (279 nm) on stainless-steel discs.

Time (min)	255 nm UV-C LED Dose (mJ/cm^2^)	255 nm UV-C LED Reduction (log PFU/mL)	279 nm UV-C LED Dose (mJ/cm^2^)	279 nm UV-C LED Reduction (log PFU/mL)
0	0	0 ^A^	0	0 ^A^
0.25	12.75	0.53 ± 0.22 ^A^	1.35	0.01 ± 0.15 ^A^
0.5	25.5	1.02 ± 0.04 ^B^	2.7	0.41 ± 0.15 ^B^
1.0	51.0	1.32 ± 0.04 ^C^	5.4	0.78 ± 0.03 ^C^
1.25	63.75	1.5 ± 0.02 ^CD^	6.75	0.98 ± 0.10 ^CD^
1.5	76.5	1.76 ± 0.05 ^D^	8.1	1.14 ± 0.09 ^D^

Capital letters depict statistically significant differences in recovered infectious titers during comparison across treatment times going down a column within one system (*p* ≤ 0.05). Data depict the averages of three replicates ± standard deviations.

**Table 3:** Inactivation of bio-aerosolized deposited HAV on glass disc surfaces by 255 nm UV-C LED or 279 nm UV-C LED systems.

Time (min)	255 nm UV-C LED Dose (mJ/cm^2^)	255 nm UV-C LED Reduction (log PFU/mL)	279 nm UV-C LED Dose (mJ/cm^2^)	279 nm UV-C LED Reduction (log PFU/mL)
0	0	0 ^A^	0	0 ^A^
0.25	12.75	0.04 ± 0.05 ^A^	1.35	0.05 ± 0.15 ^A^
0.5	25.5	0.97 ± 0.03 ^B^	2.7	0.62 ± 0.15 ^B^
1.0	51.0	1.16 ± 0.08 ^B^	5.4	0.98 ± 0.03 ^BC^
1.25	63.75	1.79 ± 0.07 ^C^	6.75	1.34 ± 0.10 ^C^
1.5	76.5	1.75 ± 0.07 ^C^	8.1	1.29 ± 0.09 ^C^

Capital/Uppercase letters depict statistically significant differences when comparing data (reduction in infectious titer) across treatment times down a column within one UV-C system (*p* ≤ 0.05). Data depict averages of three replicates ± standard deviations.

## Data Availability

The original contributions presented in the study are included in the article, further inquiries can be directed to the corresponding author.
